# Relationship between *APOE*, *PER2*, *PER3* and *OX2R* Genetic Variants and Neuropsychiatric Symptoms in Patients with Alzheimer’s Disease

**DOI:** 10.3390/ijerph20054412

**Published:** 2023-03-01

**Authors:** Susana Lozano-Tovar, Yaneth Rodríguez-Agudelo, David José Dávila-Ortiz de Montellano, Blanca Estela Pérez-Aldana, Alberto Ortega-Vázquez, Nancy Monroy-Jaramillo

**Affiliations:** 1 Facultad de Psicología, Universidad Nacional Autónoma de México (UNAM), Circuito Ciudad Universitaria Avenida, C.U., Mexico City 04510, Mexico; 2 Laboratorio de Neuropsicología Clínica, Instituto Nacional de Neurología y Neurocirugía, “Manuel Velasco Suárez”, Mexico City 14269, Mexico; 3 Departamento de Genética, Instituto Nacional de Neurología y Neurocirugía, “Manuel Velasco Suárez”, Mexico City 14269, Mexico; 4 Doctorado en Ciencias Biológicas y de la Salud, Universidad Autónoma Metropolitana, Mexico City 04960, Mexico; 5 Departamento de Sistemas Biológicos, Universidad Autónoma Metropolitana, Unidad Xochimilco, Mexico City 04960, Mexico

**Keywords:** Alzheimer’s disease, neuropsychiatric symptoms, *OX2R/HCRTR2* gene, *PER2* gene, *PER3* gene, *APOE* gene

## Abstract

Alzheimer’s disease (AD) is characterized by the presence of neuropsychiatric or behavioral and psychological symptoms of dementia (BPSD). BPSD have been associated with the *APOE_ε4* allele, which is also the major genetic AD risk factor. Although the involvement of some circadian genes and orexin receptors in sleep and behavioral disorders has been studied in some psychiatric pathologies, including AD, there are no studies considering gene–gene interactions. The associations of one variant in *PER2*, two in *PER3*, two in *OX2R* and two in *APOE* were evaluated in 31 AD patients and 31 cognitively healthy subjects. Genotyping was performed using real-time PCR and capillary electrophoresis from blood samples. The allelic-genotypic frequencies of variants were calculated for the sample study. We explored associations between allelic variants with BPSD in AD patients based on the NPI, PHQ-9 and sleeping disorders questionnaires. Our results showed that the *APOE_ε4* allele is an AD risk variant (*p* = 0.03). The remaining genetic variants did not reveal significant differences between patients and controls. The *PER3*_rs228697 variant showed a nine-fold increased risk for circadian rhythm sleep–wake disorders in Mexican AD patients, and our gene–gene interaction analysis identified a novel interaction between *PERIOD* and *APOE* gene variants. These findings need to be further confirmed in larger samples.

## 1. Introduction

Alzheimer’s disease (AD) is characterized by cognitive and behavioral symptoms, also called neuropsychiatric symptoms, or behavioral and psychological symptoms of dementia (BPSD). AD is the most common form of dementia and may contribute to 60–70% of cases [1].

BPSD have been related to worse impairments in the functional and cognitive performance of patients [2,3] and are often confused with other psychiatric diagnoses [4]. In addition, BPSD are the major cause of institutionalization of AD patients and are a major concern for their caregivers [5]. The pathogenesis of these heterogeneous groups of non-cognitive symptoms is multifactorial, involving biological, psychological and social factors [6]. BPSD include mood disorders, aggression, psychotic symptoms and behavioral problems [7]. The literature has shown differences in the prevalence rates of the most common BPSD by type of dementia and high heterogeneity of the reports [8]; for instance, two studies, including a meta-analysis, documented depression and apathy as the most common BPSD in AD patients [9,10]; meanwhile, a longitudinal study showed a high incidence of depression, anxiety, apathy and sleep disturbance in Mexican patients with AD [11].

Sleep disturbance is a common symptom in neurodegenerative diseases such as AD [12] and is one of the BPSD that alters the quality of life of patients and caregivers the most [13]. In AD, circadian cycle alteration has been found [14], including insomnia, increased total sleep time, nocturnal awakening and daytime sleepiness; these, in turn, are associated with a more accelerated progression of cognitive impairment [15,16] and also with an exacerbation of depression and altered dietary intake [17]. Sleep and circadian rhythms are intrinsically linked with several sleep traits (timing and duration), influenced by both sleep homeostasis and the circadian phase.

One of the most important processes occurring during the sleep phase is the cleaning of waste products by the glymphatic system, which, in turn, has been related to the pathogenesis of AD through amyloid-β (Aβ) deposition during nights [18]. On the other hand, sleep cycle abnormalities and behavioral and psychological symptoms in AD may influence the function of the glymphatic system. Orexins could support the proper functioning of the glymphatic system, as they have been seen to increase the removal of metabolic byproducts from the brain [19]. Additionally, alterations of the circadian cycle, a relevant feature of AD, have also been associated with changes in the glymphatic system [20]. Thus, taken together, the BPSD symptoms in AD and the circadian/orexin genes are an interesting scenario for research. 

Genetic variants in several circadian genes have been associated with diurnal preference and other sleep measurements [21]. As explained below, there are currently several neurobiological proposals to understand the association between sleep disturbance and BPSD of AD. 

The *APOE* gene has three main alleles, ε2, ε3 and ε4, which encode for their corresponding isoforms of apolipoprotein E, ApoE2, ApoE3 and ApoE4, respectively. The main isoform, ApoE3, and the minor isoform, ApoE4, have a strong affinity to the low-density lipoprotein receptor (LDLR) and have been associated with a higher risk of AD [22]. *APOE_ε4* has also been considered a risk factor for BPSD in AD [23]; for instance, Mou et al. (2015) found that the proportion of *APOE_ε4* carriers with BPSD was much higher than that of non-*APOE_ε4* carriers in a group of AD patients; their findings suggested that *APOE_ε4* may also be a risk factor for neuropsychiatric symptoms in this disease [24]. Moreover, a recent study demonstrated a synergistic effect of BPSD (depression, apathy, anxiety, agitation, appetite or irritability) with *APOE_ε4* status on conversion to dementia in a large sample of patients with mild cognitive impairment [23]. However, there are no reports that have studied interactions between *APOE* status and circadian genetic variants in AD-BPSD.

Circadian gene dysregulation is one of the proposals that has been associated with neuropsychiatric pathologies and BPSD in AD [25]. Physiological rhythmic regulation is partly exerted by the *CLOCK* genes (circadian locomotor output cycles kaput), including the *PERIOD* genes (e.g., *PER2* and *PER3*). Variants of these genes have been associated with sleep regulation and cognition [21,26], in addition to adult psychiatric pathologies such as anxiety [27], major depressive disorder [28] and depression [29]. These genes have also been related to BPSD in different types of dementia, including AD; however, further studies are needed to determine their clinical utility in dementia [30].

Perturbations in the *CLOCK* gene, including genetic variants, are associated with common psychiatric illnesses, as well as with circadian disturbances and comorbidities. For instance, a longer circadian period has been found in patients with bipolar disorder compared to controls [31], and the presence of the *PER2*_rs2304672 variant has been associated with a higher risk for bipolar disorder [32].

A variable number of tandem repeats (VNTR) in the coding region of *PER3* (rs57875989) may present four or five repeats of eighteen amino acids in the corresponding protein. The *PER3*_5/5 homozygous genotype has shown a protective effect for bipolar disorder compared to the 4/4 genotype [32], and the latter has demonstrated a predisposition to higher levels of anxiety [27]. Moreover, the *PER3*_5/5 genotype has been associated with the presence of cognitive decline and altered brain integrity in terms of structural integrity and functionality in older adults [26]. In contrast, another study found no association between *PER3* genetic variants and depression in older adults [33]. 

Another proposal to explain BPSD in AD is related to sleep disturbance and dysregulation of the orexin (OX) or hypocretin neuropeptide precursor (HCRT) system [34,35]. Davies et al. [36] hypothesized that hippocampal upregulation of neuropeptides, including orexins and their receptors (OXR), could be involved in the AD pathophysiology, since the patients have increased nocturnal activity, excessive daytime sleepiness and weight loss. There are scarce studies of the association of circadian genes in AD, and the majority of them included Asiatic or Caucasian patients [37,38,39,40,41]. Only one previous study was conducted in a Latin American population with AD, and the authors did not find associations with *PER2*, *PER3*, *CLOCK* and *OX2R* (also known as *HCRTR2*) genetic variants [33]. 

The orexin system has also been involved in the presence of BPSD in patients with AD. It has been documented that AD patients showing more BPSD with higher scores for the Neuropsychiatric Inventory (NPI) correlate with lower scores in the Mini-Mental State Examination (MMSE) screening test, together with higher levels of orexins and TAU in cerebrospinal fluid (CSF), a more altered sleep structure and an increased likelihood of nocturnal awakening compared to AD patients not affected by BPSD [42,43]. 

Taken together, these findings highlight that *APOE*, circadian genes and the orexin system may influence sleep deterioration and the occurrence of BPSD in AD. Herein, we aimed to determine the association between genetic variants of *APOE*, *PER2*, *PER3* and *OX2R* with BPSD in patients with AD and paired cognitively healthy controls.

## 2. Materials and Methods

### 2.1. Subjects

All procedures were carried out in accordance with the declaration of Helsinki. Written informed consent was obtained from all control individuals and primary caregivers on behalf of AD patients before they participated in the study. The protocol was approved by the Research and Ethics Committees of the Instituto Nacional de Neurología y Neurocirugía (project identification code INNN_11/20, date of approval May 2021). 

#### Inclusion and Exclusion Criteria

We included 62 non-related Mestizo Mexican (MM) subjects (with four Mexican-born grandparents and a maximum of one Spanish grandparent), equal or over 60 years of age, with no history of neurological or psychiatric disease and with a minimum of six years of schooling. The patients’ group consisted of 31 subjects with a clinical diagnosis of AD who were accompanied by primary caregivers. Other types of dementia were excluded. The control group consisted of 31 unrelated, cognitively healthy subjects. Additionally, 100 MM control samples were included to determine the genetic structure of the population. Consecutive AD patients were recruited from the outpatient clinic of our institution, and cognitively healthy controls were employees of the INNN or were companions/partners of the patients. Individuals were excluded if they did not meet the inclusion criteria and did not complete the evaluations.

### 2.2. Molecular Analysis

Genomic DNA was extracted from peripheral blood samples of all participants. The genotyping of *PER3*_rs228697 and rs57875989; *PER2*_rs2304672; *APOE*_rs7412 and rs429358; and *OX2R*_rs9370399 and rs2653349 variants was performed using real-time PCR on STEP ONE equipment (Thermofisher, Écublens, Switzerland). The VNTR of *PER3*_rs57875989 was determined via capillary electrophoresis on an AB3130 genetic analyzer (Applied Biosystems, Sparta, NJ, USA). Allelic and genotypic frequencies of the seven variants were determined in 100 additional MM control samples.

### 2.3. Clinical and Neuropsychological Evaluations 

A clinical diagnosis of Alzheimer’s disease according to DSM 5 criteria [44] and differential diagnoses were performed on the patients by at least two specialists in neuropsychiatry. Sociodemographic, neuropsychiatric and sleep questionnaires were applied to the participants, as explained below.

The Barthel index of activities of daily living, the Montreal Cognitive Assessment (MoCA) measurement, the Patient Health Questionnaire (PHQ-9) and a sleeping disorders questionnaire in the elderly were used to assess the general health status of the participants; specifically, the cognitive deficits and depression and sleep symptoms in patients and controls were determined. The 12-item Neuropsychiatric Inventory (NPI-12) was used to evaluate the neuropsychiatric symptoms in AD patients. These questionnaires were given to the patient’s relative or caregiver and to the controls by the neuropsychologists in a single session. A second session was required when requested by the caregiver/patient/control.

Barthel’s index of activities of daily living was used to evaluate the patient’s independence in basic activities such as bathing, eating, dressing, toileting and moving around. It indicates the degree of independence, from total to severe dependence (scoring 0 to 100) [45]. The MoCA screening test for cognitive impairment in the elderly was chosen to evaluate cognitive functions with a cutoff value of 26 [46]. The PHQ-9 multipurpose instrument was used for screening, diagnosing, monitoring and measuring the severity of depression. It has a global score with a cutoff value of four, scoring affective and somatic symptomatology [47]. The Cummings Neuropsychiatric Inventory (NPI-12) obtains information on the presence of psychopathology in patients with AD and other dementias. Ten behavioral and two neurovegetative areas are included: delusions, hallucinations, agitation/aggression, depression, anxiety, elation/euphoria, apathy/indifference, disinhibition, irritability, aberrant motor behavior, sleep and nighttime behavior disorders and appetite and eating disorders. The score for each domain was calculated as the frequency multiplied by the severity. The global NPI score was calculated by adding the scores of the 12 domains together [48].

The sleeping disorders questionnaire in the elderly identifies and evaluates the frequency of sleep disturbances associated with the elderly with or without dementia. It yields eight categories of sleep disturbance: obstructive sleep apnea (OSA), restless legs syndrome (RLS), hypersomnia, rapid eye movement (REM) sleep behavior disorder (RBD), circadian rhythm sleep–wake disorders (CRSWD), periodic limb movement disorder (PLMD) and insomnia. The higher the score, the greater the symptomatology present (i.e., 0 = never to 4 = always) [49]. After the neuropsychological evaluation, a genealogy and family history were constructed and blood draws were carried out by the geneticist in the same or in the second session for all participants.

### 2.4. Statistical Analysis

Descriptive statistics were used for clinical, sociodemographic and BPSD variables. Data for categorical variables are presented as numbers and frequencies and as mean values for continuous variables. Allelic and genotypic frequencies were assessed for all the genetic variants studied. Fisher’s exact test was used to compare the genotypic frequencies of *APOE*, *PER2*, *PER3* and *OX2R* variants for each BPSD variable. Allelic and genotypic frequencies were calculated in both groups. The Hardy–Weinberg (H-W) equilibrium and differences in the frequencies of the variants among groups were determined using a chi-square test. Statistical analyses were performed using SPSS software V 22.0 (IBM, Tokyo, Japan) and Prism for Windows ver. 5.01 (GraphPad Software, La Jolla, CA, USA). A *p*-value of <0.05 was considered statistically significant. Then, we used the nonparametric multifactorial dimensionality reduction (MDR) algorithm to model gene–gene (epistatic) interactions and the predictive power of pooled variants. The MDR algorithm evaluates all possible genetic models by reducing the dimensionality of genotype determinants and provides the best genetic model to predict outcomes. The cross-validation consistency score is a measure of the degree of consistency with which the selected model is identified as the best model among all the possibilities considered [50]. The associations were analyzed with Student’s *t*-test, Mann–Whitney U test or Fisher’s exact test depending on the data distribution.

## 3. Results

### 3.1. Sociodemographic and Clinical Characteristics

Thirty-one patients with AD and thirty-one cognitively healthy controls were evaluated. Medications and pathologies were classified according to those most frequently presented. The mean age of the patients was 73.40 ± 8.76 years, and that of the controls was 69.16 ± 8.27 years. Both groups were homogeneous and comparable in age, gender, schooling and pathologies presented (Table 1) (*p* > 0.05). There were 74.2% of patients who had late-onset AD; the remaining had early-onset AD, with an average of 5.5 ± 3.4 years of evolution of the disease at the sampling time, and were under antidementia drugs for at least six months prior to their enrollment. 

### 3.2. Description of BPSD in Patients with AD and Cognitively Healthy Controls

The presence of BPSD in patients with AD for each symptom, according to NPI and PHQ-9 scores, is presented in percentages in Supplementary Table S1. In our sample, all patients showed two or more BPSD symptoms. Regarding the NPI, the symptom with the highest percentage was apathy (84%), followed by anxiety (81%), irritability (75%) and depression (72%); meanwhile, affective symptomatology was the most observed symptom in the patients on the basis of PHQ-9 scores.

Regarding sleep disturbances, evaluated with the sleeping disorders questionnaire in the elderly, insomnia and OSA were present in all patients and controls. In the patients’ group, other symptoms observed were CRSWD (87.5%) and parasomnia (84.4%); meanwhile, in the group of controls, hypersomnia (87.1%) and CRSWD (77.4%) occupied the third and fourth most frequent symptoms (Supplementary Table S2).

### 3.3. Allelic and Genotypic Frequencies of the Genetic Variants Studied

The allelic and genotypic frequencies of the *PER2*, *PER3*, *OX2R* and *APOE* variants analyzed were calculated (Supplementary Table S3). All the genetic variants were found to be in H-W equilibrium, as calculated with the chi-square test in 100 MM controls. The frequencies of the alternative alleles for almost all of the variants analyzed were similar between both groups (controls/patients; *p* > 0.05) as follows: *PER2*_rs2304672 *f*(C) = 0.03/0.05, *PER3*_rs228697 *f*(G) = 0.05/0.11, *PER3*_rs57875989 *f*(5 repeats) = 0.18/0.19 and *OX2R*_rs9370399 *f*(C) = 0.27/0.37 and rs2653349 *f*(G) = 0.97/0.94. The exception was the *APOE_ε4* allele, which showed a significant difference between both groups; *f*(ε4) = 0.08/0.16, *p* = 0.03 (Supplementary Table S3).

The observed frequencies of the genetic variants included were similar to those previously reported in the international databases: *PER2*_rs2304672, *f*(G) = 0.94, *f*(C) = 0.06; *PER3*_rs57875989, *f*(4 repeats) = 0.84, *f*(5 repeats) = 0.16; *PER3*_rs228697, *f*(G) = 0.06, *f*(C) = 0.94; *APOE*_rs7412, rs429358, *f*(ε3) = 0.88, *f*(ε4) = 0.04; *OX2R*_rs9370399, *f*(A) = 0.65, *f*(C) = 0.35; and *OX2R*_rs2653349, *f*(A) = 0.14, *f*(G) = 0.86 (dbSNP, NCBI). The detailed characteristics of all genetic variants included in the study are found in Table S4. 

### 3.4. Associations between Allelic Variants with BPSD in Patients with AD based on NPI, PHQ-9 and Sleeping Disorders Questionnaire Evaluations

BPSD in the patients’ group were evaluated with NPI, PHQ-9 and the sleeping disorders questionnaire; then, they were analyzed for associations with the genetic variants of *PER2*, *PER3*, *OX2R* and *APOE*. This analysis identified the following genetic variants as associated with BPSD: Anxiety symptoms showed a significant association with the *APOE_ε4* allele (*p* = 0.029) when evaluated by the NPI scale. The *OX2R*_rs9370399 variant was associated with hypersomnia (*p* = 0.046) and circadian rhythm disorder (*p* = 0.031), whereas the *PER3*_rs228697 variant was associated with circadian rhythm disorder (*p* = 0.028) when using the sleeping disorders questionnaire (Table 2). Regarding the evaluations of the PHQ-9 scale, no associations were observed for total, somatic or affective depressive symptomatology (*p* > 0.05). The odds ratios were calculated for those statistically significant variables (Table 2). Only the *PER3*_rs228697 variant persisted and showed a high risk for CRSWD symptoms in patients with AD (OR = 9.736). 

### 3.5. Gene–Gene Interaction Analysis by MDR

**Figure 1:**
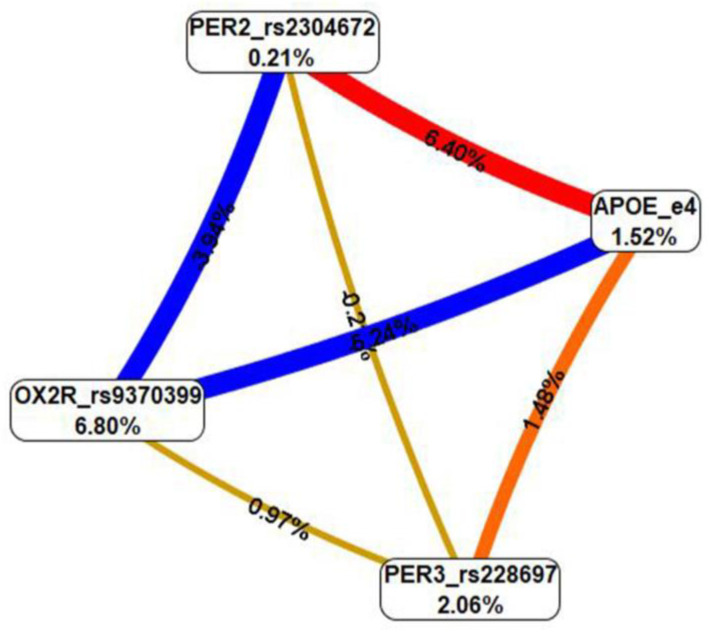
Interaction entropy graph for gene–gene interactions via multifactor dimensionality reduction (MDR) analysis. Information gain summary by main effects can be found inside the boxes. The effects by pairs indicate interaction in red or orange lines, which can be interpreted as a synergistic or non-additive relationship; meanwhile, negative entropy (yellow-green or green lines) indicates independence or additivity (redundancy). The best model in gene–gene interaction was *PER2*_rs2304672 and *PER3*_rs228697 with *APOE_ε4*. This result suggested that the interaction between the *PER2*-*PER3* and *APOE_ε4* genes may play an important role in the pathogenesis of BPSD in our sample of AD patients.

Based on MDR analysis, the best model included the allelic variants *PER2*_rs2304672, *PER3*_rs228697 and *APOE_ε4* and had a CV of 5/10. This model presented a *p*-interaction value of 0.0025. The highest interaction presented was obtained for the *PER2*_rs2304672 and *APOE_ε4* variants with a gain information value of 6.40%, followed by the interaction between *PER3*_rs228697 and *APOE_ε4* allelic variants with a gain information value of 1.48% (Figure 1).

In order to carry out protein–protein interactions between the corresponding gene products, we used the STRING tool, a protein–protein interaction network functional enrichment analysis [51]. A direct interaction between PER2 and PER3 was confirmed. Then, an indirect interaction between the mentioned PERIOD proteins with *APOE* was mediated by SIRT1 (Figure S1). Sirtuin 1 is an enzyme that deacetylates transcription factors that contribute to cellular regulation (reaction to stressors and longevity).

### 3.6. Potential Synergistic Associations between APOE_ε4 Carrier Status Plus Multilocus Genotype with BPSD in the AD Progression of Patients

In order to estimate whether the variants of the circadian genes plus the presence of the *APOE_ε4* allele were associated with faster AD progression, patients were grouped according to their *APOE_ε4* carrier status (carriers vs. non-carriers). They were also grouped by a multilocus genotype. This genotype was based on the presence of alternative alleles in the genetic variants: rs228697, rs57875989, rs265334 and rs9370399 (i.e., alternative multilocus genotype vs. wildtype multilocus genotype). Then, possible associations of synergistic genotypes (*APOE_ε4* status and circadian variants) for BPSD in AD progression (depression, sleep disorder, delusions, hallucinations and anxiety) were explored. No associations were found.

## 4. Discussion

In the present study, we expanded the information about frequencies of BPSD in Mexican patients with AD and their association with *APOE*, *PER2*, *PER3* and *OX2R* gene variants in both AD patients and cognitively healthy controls. 

Our sample (patients and controls) predominantly received medium and high schooling, representing a higher level of schooling than the data reported at the national level. This may be related to the fact that our institution is a third-level health care center that offers specialized care for patients with neurodegenerative disorders; hence, all of our AD patients had additional chronic pathologies that were under treatment and properly managed. The most frequent chronic pathologies found in both groups were of the vascular type, including diabetes and arterial hypertension, results which were expected according to epidemiological data of the Mexican population [52]. 

Regarding BPSD in AD patients, apathy was identified as the most frequent symptom, unlike what was previously reported in the Mexican population [11] but similar to what was found in a meta-analysis [10]. Anxiety ranked second, equal to what was described in the literature. Irritability, a symptom that has been reported at a low frequency in the natural course of AD [10], appeared as the symptom with the third-highest frequency and severity. This could be due to the cognitive and functional deterioration of the patients. Patients with depression presented higher scores on the NPI scale and in the eight categories of the sleep disorder questionnaire. The above data may be associated with depression, one of the most prevalent symptoms in AD [52]. The highest percentage of sleep disturbances, present in both groups, appeared for insomnia and OSA, which are common symptoms for the Mexican older adult population with or without dementia [53]. Symptoms such as circadian cycle disturbance, parasomnia and hypersomnia were more prevalent in the patient group, as expected in AD [16,54].

The genotypic and allelic frequencies of the MM sample were similar to those previously documented in the international databases. The comparison of allelic and genotypic frequencies between AD patients and controls confirmed the *APOE_ε4* allele as the main genetic variant risk for the development of AD (*p* = 0.029) (Table 2). The remaining genetic variants did not show significant differences between the study groups. Therefore, the interactions of BPSD with gene variants were analyzed among AD patients. We found an association between anxiety and the *APOE*_*ε4* allele, using the NPI scale. Of note, this has been previously correlated in animal models [5] and patients [23]. Animal studies have suggested that the relationships between *ApoE* genotypes and anxiety may be mediated by changes in neurons in the amygdala [55]. A higher frequency of the *OX2R*_rs9370399 variant in the patients´ group with circadian rhythm disorder and hypersomnia was observed. Interestingly, this last association has been previously proposed during a major depressive episode of bipolar disorder, based on a genome-wide association (GWAS) analysis [56]. Circadian cycle disturbance is one of the most common sleep disturbances in AD and is related to *CLOCK* genes; however, little is known about how they interact to change the course of AD [57]. In our AD patients, the *PER3*_rs228697 variant was associated with symptoms of circadian rhythm disorder according to the sleeping disorders questionnaire. Circadian rhythm disorders aggravate the deposition of amyloid plaques in the brains of AD patients. Therefore, improving the circadian rhythm of AD patients may slow down the pathological development of neurodegeneration [58]. 

The gene–gene interaction analysis by MDR included *PER2*_rs2304672, *PER3*_rs228697 and *APOE_*ε4 variants in the best model. There have been previous reports documenting genetic interactions for eveningness or diurnal phenotypes among *PERIOD* and other circadian genes in Korean and Brazilian populations, respectively [59,60]. There is also evidence of an association between sleep quality and *APOE_ε4* in healthy older adults [61], with an increased risk of insomnia [62] and obstructive sleep apnea/sleep-disordered breathing in both adults [63,64] and children [65]. Recently, *APOE_ε4* homozygosity was associated with sleep disturbance, independent of AD pathological change and clinical functional status in individuals with and without dementia [66]. It has been hypothesized that the presence of the *APOE_ε4* allele instigates entry into a feed-forward loop, where sleep problems increase Aβ deposition (or reduce Aβ clearance via impaired circulation) in the brain, which then further disrupts sleep brain circuitry [61]. Recently, it was postulated that *APOE_ε4* affects sleep by mechanisms that are independent of AD pathological change [66]. It is known that glymphatic impairment caused by sleep disturbance results in Aβ aggregation and increased risk of AD [18,19,20]. Therefore, future research should focus on glymphatic dysfunction at the molecular genetic level as a potential bridge between sleep disorders and other BPSD in AD.

This is the first time that a *PER2-PER3-APOE_ε4* interaction model has been reported in AD patients presenting with BPSD. Interestingly, that same *PER3* variant showed a high risk for circadian rhythm sleep–wake disorders in the patients´ group (OR = 9.736). A preceding report found that the *APOE* genotype and *CLOCK_*T3111C variant seem to interact with cardiovascular risk factors in patients with cognitive impairment to influence the progression to AD [38]. It would be relevant for future research to investigate the impact of the *APOE* genotype on the circadian system and sleep–wake homeostasis and the way they interact in defining sleep and waking cognition in AD patients to drive the onset and progression of this disease.

Synergistic interactions between BPSD and *APOE_ε4* have been identified among patients with mild cognitive impairment when predicting incident dementia [23]. The analysis of *APOE_ε4* carrier status plus the alternative multilocus genotype with the following BPSD as indicators of AD progression (depression, sleep disorder, delusions, hallucinations and anxiety) did not find any association in this population of AD patients. This could be due to the small sample sizes of our subgroups and should be explored in future studies with larger samples. Taking into account past and present findings, the combination of BPSD, *APOE* carrier status and circadian genotypes could be a useful strategy to identify the most vulnerable patients with cognitive impairments to dementia conversion and also to apply early psychological interventions based on genetic findings in AD patients that present with specific BPSD. 

One limitation of the study was the small sample size of participants; moreover, we did not include environmental risk factors or other relevant circadian gene variants, such as *BMAL1*, *PER1* and *CRY1/2*, which activate *PERIOD* transcription, and sleep disturbances were subjectively evaluated; therefore, these results should be considered to be preliminary and need to be confirmed in larger samples. One of the strengths of our study was that we included AD patients who presented with BPSD, with a clinical diagnosis confirmed by specialists in dementia. Patients and controls were matched by sex, age, ethnicity, level of schooling and even by concomitant health conditions. Another strength of the present study was the use of a validated and specific scale to assess sleep disturbances in older adults with and without dementia. Most scales to assess sleep are not focused on the elderly, whose sleep disturbance characteristics are different from those of the adult population. 

Despite the numerous efforts to counteract this neurodegenerative disorder, no therapies have so far been proven to prevent AD onset or progression. There is an urgent need to find more valuable biomarkers to delay/modify the progression of BPSD in AD. Currently, orexins are being studied as a therapeutic target for the treatment of AD, considering not only sleep disturbances but also their interactions with the Aβ and TAU proteins (key actors in the AD pathophysiology) [54,67]. In this context, further research involving larger sample sizes, including a group of AD patients without BPSD, and exploring other circadian genes will provide more information on possible associations and interactions between these genes and particular mechanisms of BPSD in AD.

## 5. Conclusions

The *PER3*_rs228697 variant showed a nine-fold increased risk for CRSWD in Mexican AD patients, and this risk may be even higher in those patients who also carry the *APOE_ε4* allele due to a potential *PERIOD–APOE* interaction. 

## Figures and Tables

**Table 1:** Description of sociodemographic and clinical characteristics of patients with AD (n = 31) and controls (n = 31).

Characteristic		Patients		Controls	
		(n = 31)		(n = 31)	
		Frequency	%	Frequency	%
Gender	Female	15	49	16	52
	Male	16	51	15	48
Education	Elementary/Middle school	10	32	9	29
	High school	8	26	11	35
	University	13	42	11	36
Comorbidities	None	12	39	10	32
	Vascular ^1^	8	26	9	29
	High cholesterol	1	3	1	3
	Multiple pathologies ^2^	8	26	4	13
	Other	2	6	7	23
Medication ^4^	Antidementia drugs	-	69	-	0
	Vascular	-	50	-	36
	SSRIs/Antipsychotic	-	66	-	3
	Polypharmacy ^3^	-	53	-	19
	Antipsychotic and Antidementia drugs	-	57	-	0
Dependence level	Total/Complete	2	6	0	0
	Severe	3	10	0	0
	Moderate	4	13	0	0
	Low	15	48	0	0
	Independent	7	23	31	100
Age (in years)		ME	SD	ME	SD
		73.40	8.76	69.16	8.27
MoCA score		6.78	6.20	26.81	1.85
Age at onset of AD (years)		67.5	8.96	NA	NA

^1^ Including diabetes and high blood pressure; ^2^ presence of more than three chronic diseases; ^3^ more than three drugs consumed simultaneously; ^4^ all patients were being treated with one or more drugs, and hence, the percentages in “medication” are not exclusive and add up to more than 100%. SSRIs: selective serotonin reuptake inhibitors; ME: mean value; SD: standard deviation; NA: not applicable.

**Table 2:** Associations of allelic variants and BPSD according to NPI and PHQ-9 scale evaluations in AD patients (n = 31).

		Allelic Variants											
		rs9370399 (*f* = 0.37)		rs2653349 (*f* = 0.94)		rs2304672 (*f* = 0.05)		rs57875989 (*f* = 0.19)		rs228697 (*f* = 0.11)		rs429358 ^⚕^ (*f* = 0.16)	
		AAF	*p* Value	AAF	*p* Value	AAF	*p* Value	AAF	*p* Value	AAF	*p* Value	AAF	*p* Value
PHQ-9	Depression	0.369	1.00	0.913	1.000	0.956	1.000	0.195	1.000	0.108	1.000	0.195	1.000
NPI	Delusions	0.368	0.597	0.941	1.000	0.941	1.000	0.147	0.349	0.088	1.000	0.264	0.192
	Hallucination	0.333	0.788	0.958	1.000	0.958	1.000	0.208	1.000	0.125	0.669	0.208	1.000
	Agitation	0.357	0.784	0.952	0.588	0.928	0.545	0.190	1.000	0.142	0.164	0.190	1.000
	Depression	0.369	0.778	0.954	0.573	0.954	1.000	0.159	0.305	0.113	0.662	0.204	1.000
	Anxiety	0.400	0.508	0.920	0.578	0.960	0.482	0.200	1.000	0.100	1.000	0.140	0.029 *
	Euphoria	0.350	1.000	0.900	0.588	0.900	0.241	0.200	1.000	0.200	0.079	0.150	0.735
	Apathy	0.384	0.731	0.942	0.515	0.942	1.000	0.173	0.391	0.115	0.577	0.173	0.391
	Disinhibition	0.369	1.000	0.925	1.000	0.925	0.546	0.175	0.740	0.125	0.449	0.200	1.000
	Irritability	0.413	0.369	0.913	0.565	0.934	0.562	0.152	0.268	0.130	0.325	0.2174	0.714
	Aberrant motor behavior	0.361	1.000	0.944	1.000	0.916	0.258	0.111	0.101	0.115	0.689	0.194	1.000
	Sleep	0.404	0.575	0.928	1.000	0.952	1.000	0.238	0.306	0.119	0.654	0.142	0.177
	Appetite	0.369	0.778	0.977	0.70	0.954	1.000	0.181		0.090	1.000	0.227	0.481
Sleeping Disorders Questionnaire	Insomnia	0.409	0.396	0.955	0.313	0.955	1.000	0.227	0.481	0.113	0.662	0.159	0.305
	OSA	0.404	0.575	0.928	1.000	0.952	1.00	0.190	1.000	0.142	0.164	0.166	0.500
	RSL	0.500	0.425	0.750	0.077	0.923	1.000	0.125	1.000	0.250	0.168	0.250	0.642
	Hypersomnia	0.230	0.046 *	1.000	0.132	0.961	0.567	0.230	0.528	0.192	0.074	0.153	0.746
	RBD	0.500	0.661	1.000	1.000	1.000	1.000	0.166	1.000	0.166	0.472	0.333	0.328
	Parasomnia	0.346	0.794	1.000	1.000	0.961	1.000	0.192	1.000	0.153	0.227	0.192	1.000
	CRSWD	0.208	0.031 *	1.000	0.151	0.958	1.000	0.250	0.511	0.208	0.028 *	0.166	0.670
	PLMD	0.333	1.000	1.000	1.000	1.000	1.000	0.166	1.000	0.166	0.472	0.333	0.328
Genetic association													
NPI	Genetic Variant		*p* Value		OR Value		CI 95%						
Anxiety	rs429358		0.029		0.223		0.056–0.923						
Sleeping Disorders Questionnaire													
Hypersomnia	rs9370399		0.046		0.335		0.109–1.030						
CRSWD	rs9370399		0.031		0.292		0.090–0.945						
	rs228697		0.028		9.736		1.060–89.398						

OSA: obstructive sleep apnea; RSL: restless legs syndrome; RBD: rapid eye movement (REM) sleep behavior disorder; CRSWD: circadian rhythm sleep–wake disorders; PLMD: periodic limb movement disorder; OR: odds ratio; AAF: alternative allelic frequency; ^⚕^ the *APOE_ε4* allele is defined by the rs429358 variant; *f*: frequency; CI: confidence interval; * *p* < 0.05. NPI: Neuropsychiatric Inventory; PHQ-9: Patient Health Questionnaire.

## Data Availability

The data presented in this study are available in the Supplementary Materials [pending link at https://www.mdpi.com/article/10.3390/ijerph20054412/s1].

## Supplementary Materials

The following supporting information can be downloaded at: https://www.mdpi.com/article/10.3390/ijerph20054412/s1, Table S1: Presence of BPSD (in percentage) in patients with AD according to NPI and PHQ-9 scales; Table S2: Presence of sleep disorders (in percentage) in AD patients and controls according to the scale of quality of sleep disorders in the elderly; Table S3: Allelic and genotypic variant of patients and controls; Table S4: Characteristics of genetic variants included in the study; Figure S1: Protein-protein interactions of PERIOD 2 and 3 with APOE. This indirect interaction is mediated by SIRTUIN1. References [22,27,56,67,68,69,70,71,72,73,74,75,76,77,78,79,80] are cited in the supplementary materials.
