# Supplementary material for: Relationship between APOE, PER2, PER3 and OX2R Genetic Variants and Neuropsychiatric Symptoms in Patients with Alzheimer’s Disease
Source: Int J Environ Res Public Health. 2023 Mar 1;20(5):4412. doi: 10.3390/ijerph20054412 (PMC10001852; doi:10.3390/ijerph20054412)
Supplement: Supplementary file 1 [file ijerph-20-04412-s001.zip › ijerph-2184456-supplementary.pdf]

Supplementary Table S1. Presence of BPSD (in percentage) in patients with AD according to NPI and PHQ-9 scales.

Scale	Patients (n=31)	Frequency (%)
PHQ-9	Affective	91
	Somatic	84
NPI	Apathy	84
	Anxiety	81
	Irritability	75
	Depression	72
	Appetite	72
	Agitation	69
	Sleep	69
	Disinhibition	66
	Aberrant motor behavior	59
	Delusions	56
	Hallucinations	38
	Euphoria	31

Supplementary Table S2. Presence of sleep disorders (in percentage) in AD patients and controls according to the Sleeping Disorders Questionnaire.

Sleep disorder	Patients (n=31)	Controls (n=31)
	%	%
OSA¥	100.00	100.00
RLS¥	56.30	67.70
Hipersomnia	81.30	87.10
RBD¥	75.00	67.70

Parasomnia	84.40	67.70
CRSWD¥	87.50	77.40
PLMD¥	56.30	58.10
Insomnia	100.00	100.00

¥OSA: Obstructive sleep apnea; RSL: restless legs syndrome; RBD: Rapid Eye Movement (REM) sleep behavior disorder; CRSWD: Circadian rhythm sleep-wake disorders; PLMD: periodic limb movement disorder.

Supplementary Table S3. Allelic and genotypic variant of patients and controls

Genetic variant	Controls/Patients Allelic frequencies	p	Genotypic frequencies	p
PER3				
rs228697_C>G	C= 0.95/0.88<br>G= 0.05/0.11	0.30	CC= 0.95/0.77<br>CG= 0.04/0.22<br>GG= 0.00/0.00	0.30
rs57875989_4 or 5 repeats	4= 0.82/0.80<br>5= 0.18/0.19	0.82	4/4= 0.64/0.65<br>4/5= 0.36/0.32<br>5/5= 0.00/0.03	1.00
PER2				
rs2304672_G>C	G= 0.97/0.03<br>C= 0.03/0.05	0.65	GG= 0.93/0.90<br>GC= 0.06/0.09<br>CC= 0.00/0.00	1.00
OX2R				
rs9370399_A>C	A= 0.73/0.63<br>C= 0.27/0.37	0.08	AA= 0.45/0.35<br>AC= 0.54/0.54<br>CC= 0.00/0.10	0.11
rs2653349_A>G	A= 0.03/0.06<br>G= 0.97/0.94	0.17	AA= 0.00/0,03<br>AG= 0.06/0.06<br>GG= 0.94/0.90	1.00
APOE				
rs7412_C>T and rs429358_T>C	Ɛ3= 0.92/0.84<br>Ɛ4= 0.08/0.16	0.03*	Ɛ3/Ɛ3= 0.84/0.72<br>Ɛ4/Ɛ4= 0.16/0.28<br>Ɛ3/Ɛ4= 0.00/0.00	0.25

*p-value <0.05

Supplementary Table S4. Characteristics of genetic variants included in the study.

Variant[¥]	Location & nucleotide change	Amino acid change	Previous relevant associations
APOE gene			
rs429358	Chr19-44908684<br>NM_000041.4<br>Exon4 ǀ c.388T>C	p.Cys130Arg	Three haplotypes (ε2(388 T–526 T), ε3(388 T-526C), ε4(388C-526C)) and six genotypes (ε2/ε2, ε2/ε3, ε2/ε4, ε3/ε3, ε3/ε4, ε4/ε4) can be formed by these SNPs [68]. *APOE* gene has three main alleles, ε2, ε3 and ε4, coding for three corresponding isoforms of apolipoprotein E: E2, E3 and E4, respectively.<br>The main isoform E3 and the minor isoform E4 of APOE protein have strong affinity to Low Density Lipoprotein Receptor (LDLR) and have been associated with a higher risk of Alzheimer's disease (AD) [22]. On the contrary, E2 isoform has been described as neuroprotective, and presents low affinity for the LDL receptor [69].
rs7412	Chr19-44908810<br>NM_000041.4<br>Exon4 ǀ c.526C>T	p.Arg176Cys	
PER2 gene			
rs2304672	Chr2-239186577-239186601<br>NM_022817.3 ǀ<br>ENST00000254657<br>5´UTRǀ c.-12C>G ǀ	NA	*PER2* gene is a member of the Period family of genes and is expressed in a circadian pattern in the suprachiasmatic nucleus, the primary circadian pacemaker in the mammalian brain. This variant is associated with diurnal preference in a young Korean population [70]. It has also been associated with psychiatric illnesses involving reward dysfunction [71] and sleep disturbances [72].

PER3 gene			
rs228697	chr1-7887579<br>NM_001377275.1<br>Exon17 | c.2590C>G	p.Pro864Ala	Individuals homozygous for the *PER3* SNP rs228697 have been described with a significantly higher anxiety [27].
rs57875989	Chr1-7829993 inframe deletion. (-;GCTCTGTCCACAGGATCGCCTCCCATGAAGAATCCATCCCATCCTACTGCCAGC) Exon 18. NM_001289861.1:c.3002-13. ENST00000377532.3	p.Ala1016_Ser1033del	The number of repeats of 18 amino acids in positions 966 to 1055 is polymorphic and varies among at least two different alleles. Alleles corresponding in size to a *PER3*_4 and 5 repeats have been described. Homozygosity for *PER3*_5/5 is more likely to show morning preference, whereas homozygosity for the *PER3*_4/4 associates with evening preferences. *PER3*_5/5 homozygous show vulnerability to sleep loss with a greater cognitive decline in response to total sleep deprivation [73–78]. The *PER3* VNTR (rs57875989) has been significantly associated with depression in patients with mood disorders [22].
OX2R/HCRTR2 gene			
rs9370399	chr6-55180227<br>NM_001384272.1<br>intron variant | c.223+5417A>G	NA	The *OX2R* gene has been considered a genetic risk factor for AD [67]. It is a candidate gene in sertraline-associated insomnia in depressed patients [79], and its genetic variants have also been associated with self-reporting of being a morning person [80].
rs2653349	chr6-55277539<br>NM_001384272.1<br>Exon 5 | c.922A>G	p.Ile308Val	The *OX2R* gene has been considered a genetic risk factor for AD. Thus, the carriage of the G allele was associated with an increased AD risk (OR=2.53; CI95%=1.10-5.80) [56]. A genome-wide functional enrichment pathway analysis has revealed that this gene may be associated with hypersomnia symptoms of bipolar depression [56].

¥NCBI database of genetic variation, dbSNP. SNP, single nucleotide polymorphism. NA, not applicable. Additional information was obtained at Varsome and Franklin databases.

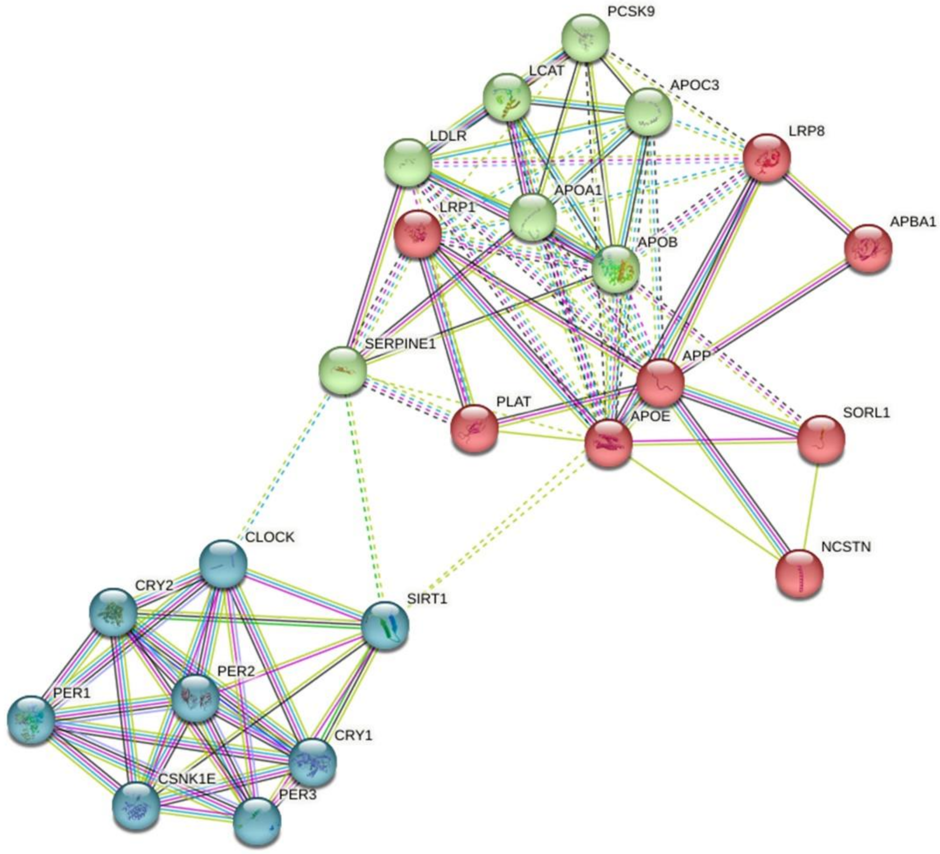

Figure S1. Protein-protein interaction of PERIOD2 and PERIOD3 with APOE. This indirect interaction is mediated by SIRTUIN1.
